# Purification and characterisation of the fission yeast Ndc80 complex

**DOI:** 10.1016/j.pep.2017.05.002

**Published:** 2017-07

**Authors:** Yuzy Matsuo, Sebastian P. Maurer, Thomas Surrey, Takashi Toda

**Affiliations:** a Cell Regulation Laboratory, The Francis Crick Institute, 44 Lincoln's Inn Fields, London WC2A 3LY, UK; b Synthetic and Systems Biochemistry of the Microtubule Cytoskeleton Laboratory, The Francis Crick Institute, 1 Midland Road, London, NW1 1AT, UK; c Centre for Genomic Regulation (CRG), Barcelona Institute of Science and Technology (BIST), Dr. Aiguader 88, 08003 Barcelona, Spain; d Universitat Pompeu Fabra (UPF), Barcelona, Spain; e Hiroshima Research Center for Healthy Aging (HiHA), Department of Molecular Biotechnology, Graduate School of Advanced Science of Matter, Hiroshima University, 1-3-1 Kagamiyama, Higashi-Hiroshima 739-8530, Japan

**Keywords:** Dis1, Fission yeast, Kinetochore, Ndc80 complex, Microtubule, TIRF microscopy

## Abstract

The Ndc80 complex is a conserved outer kinetochore protein complex consisting of Ndc80 (Hec1), Nuf2, Spc24 and Spc25. This complex comprises a major, if not the sole, platform with which the plus ends of the spindle microtubules directly interact. In fission yeast, several studies indicate that multiple microtubule-associated proteins including the Dis1/chTOG microtubule polymerase and the Mal3/EB1 microtubule plus-end tracking protein directly or indirectly bind Ndc80, thereby ensuring stable kinetochore-microtubule attachment. However, the purification of the Ndc80 complex from this yeast has not been achieved, which hampers the in-depth investigation as to how the outer kinetochore attaches to the plus end of the spindle microtubule. Here we report the two-step purification of the fission yeast Ndc80 holo complex from bacteria. First, we purified separately two sub-complexes consisting of Ndc80-Nuf2 and Spc24-Spc25. Then, these two sub-complexes were mixed and applied to size-exclusion chromatography. The reconstituted Ndc80 holo complex is composed of four subunits with equal stoichiometry. The complex possesses microtubule-binding activity, and Total Internal Reflection Fluorescence (TIRF)-microscopy assays show that the complex binds the microtubule lattice. Interestingly, unlike the human complex, the fission yeast complex does not track depolymerising microtubule ends. Further analysis shows that under physiological ionic conditions, the Ndc80 holo complex does not detectably bind Dis1, but instead it interacts with Mal3/EB1, by which the Ndc80 complex tracks the growing microtubule plus end. This result substantiates the notion that the Ndc80 complex plays a crucial role in establishment of the dynamic kinetochore-microtubule interface by cooperating with chTOG and EB1.

## Highlights

• The Ndc80 complex is a conserved outer kinetochore complex.
• The fission yeast Ndc80 complex is purified from bacteria through a two-step purification scheme.
• The Ndc80 complex possesses microtubule-binding activity similar to those from other species.
• TIRF-microscopy assays show that the complex binds the microtubule lattice.
• The Ndc80 complex tracks the microtubule plus end when mixed with Mal3/EB1.

## 1 Introduction

The kinetochore is a huge proteinaceous structure formed around the centromere on the chromosome that comprises dozens of constitutive core subunits and transient regulatory components [1]. The structure and composition of the kinetochore are subject to regulation by diverse internal and external cues, as its organisation undergoes drastic remodelling during the cell cycle and upon progression into various developmental stages [2], [3]. The key, perhaps the most critical, role for the kinetochore involves its attachment to the spindle microtubules (MTs), thereby ensuring equal partition of the genetic material to two daughter cells.

MTs are highly dynamic tubulin polymers with an intrinsic structural polarity. MTs undergo spontaneous transitions from growing to shrinking phases, a process called dynamic instability [4]. This intrinsic dynamic property of the MT underlies a wide variety of MT-mediated cellular events; e.g. the MT plus end associates with the kinetochore through a ‘search-and-capture’ mechanism, followed by deploymerisation that drives poleward movement of chromosomes. Although some of the outer kinetochore components behave as microtubule-associated proteins (MAPs) both *in vivo* and *in vitro*, inside a cell a cohort of MAPs regulate MT dynamics in a spatiotemporal manner [5].

Recent advances in the field of the kinetochore research have uncovered that the conserved outer kinetochore complexes, collectively called the KMN network (KNL1 complex-Mis12 complex-Ndc80 complex) provide direct binding platforms for the dynamic plus ends of spindle MTs [6], [7], [8], [9], [10]. In particular, the Ndc80 complex composed of Ndc80 (also called Hec1), Nuf2, Spc24 and Spc25, comprises a major, if not sole, interacting pad for MTs. It has further been shown that within this complex, Ndc80 is responsible for the MT binding activity of the complex by forming a sub-complex with Nuf2 [11], [12]. The Ndc80 complex from several organisms including budding yeast, worm and human, was produced and purified for *in vitro* studies after recombinant expression in either bacteria or insect cells. This approach has provided valuable information as to how this complex binds MTs, interacts with other proteins and is regulated by post-translational modifications such as phosphorylation and dephosphorylation [6], [8], [10], [13], [14], [15]. Although the overall rod-like architecture of the Ndc80 complex with globular domains at each end (∼57 nm in length) is well conserved across the species [6], [16], [17], some noticeable differences were observed in terms of the properties of MT binding. This includes the interaction mode between the Ndc80 complex and the MT lattice in human, worm and yeast complexes [9], [11], [12], [15], [18], [19], [20], [21]. Furthermore, while both budding yeast and human complexes were sufficient at least *in vitro* to produce dynamic, load-bearing attachments to both polymerising and depolymerising MT ends [19], only the human complex, but not the budding yeast complex, can track disassembling microtubule ends and simultaneously modulate MT dynamics [15], [20], [21].

In fission yeast *Schizosaccharomyces pombe*, like for other organisms, it is well established that the Ndc80 complex is essential for cell division and plays a central role in kinetochore-spindle MT attachment [22], [23], [24], [25]. Furthermore, as in budding yeast and human cells, fission yeast Ndc80 acts as a structural platform in the spindle assembly checkpoint signalling pathway by recruiting the MPS1/Mph1 kinase to the outer kinetochore when it is not attached to the MTs [26], [27], [28], [29], [30], [31]. Our recent studies have unveiled that Ndc80 directly or indirectly interacts with a group of MAPs, including a MT polymerase Dis1/chTOG and the plus-end binding protein Mal3/EB1 [23], [32]. Ndc80 also binds the TACC homologue Alp7, thereby loading Alp14/chTOG and the Klp5/Klp6 kinesin-8 molecules to the kinetochore [33], [34].

To dissect complex molecular pathways leading to proper kinetochore-spindle MT attachment and poleward chromosome movement, the development of *in vitro* systems composed of defined components is indispensable. The purification of a number of fission yeast MAPs required for kinetochore capture has been achieved and the *in vitro* characterisation of these proteins has been reported [32], [35], [36], [37]. By contrast, the Ndc80 complex from fission yeast has not been purified yet. Purified Ndc80 complex will be useful for the study on the interaction interface between the kinetochore and MTs. As a first step towards this goal, we report in this study the purification of the fission yeast Ndc80 holo complex from bacteria and describe its MT-binding characteristics and interaction with Mal3 as observed by Total Internal Reflection Fluorescence (TIRF) microscopy-based *in vitro* reconstitution assays.

## 2 Materials and methods

### 2.1 Protein production and purification of *S. pombe* Ndc80 holo complex

Nucleotide fragments of *ndc80, nuf2, spc24 and spc25* cDNA (full-length 624, 441, 198, 238 a.a) that were codon optimised for *E. coli* were synthesised (GeneArt Gene Synthesis) and subcloned into the pET Duet-1 (Novagen) or its derivative vector, in which a Z-tag, a *Staphylococcus aureus* protein A-binding affinity tag [38], [39] or a oligo-histidine containing Z-tag (6His-Z) was added to pET Duet-1.

**Fig. 1:**
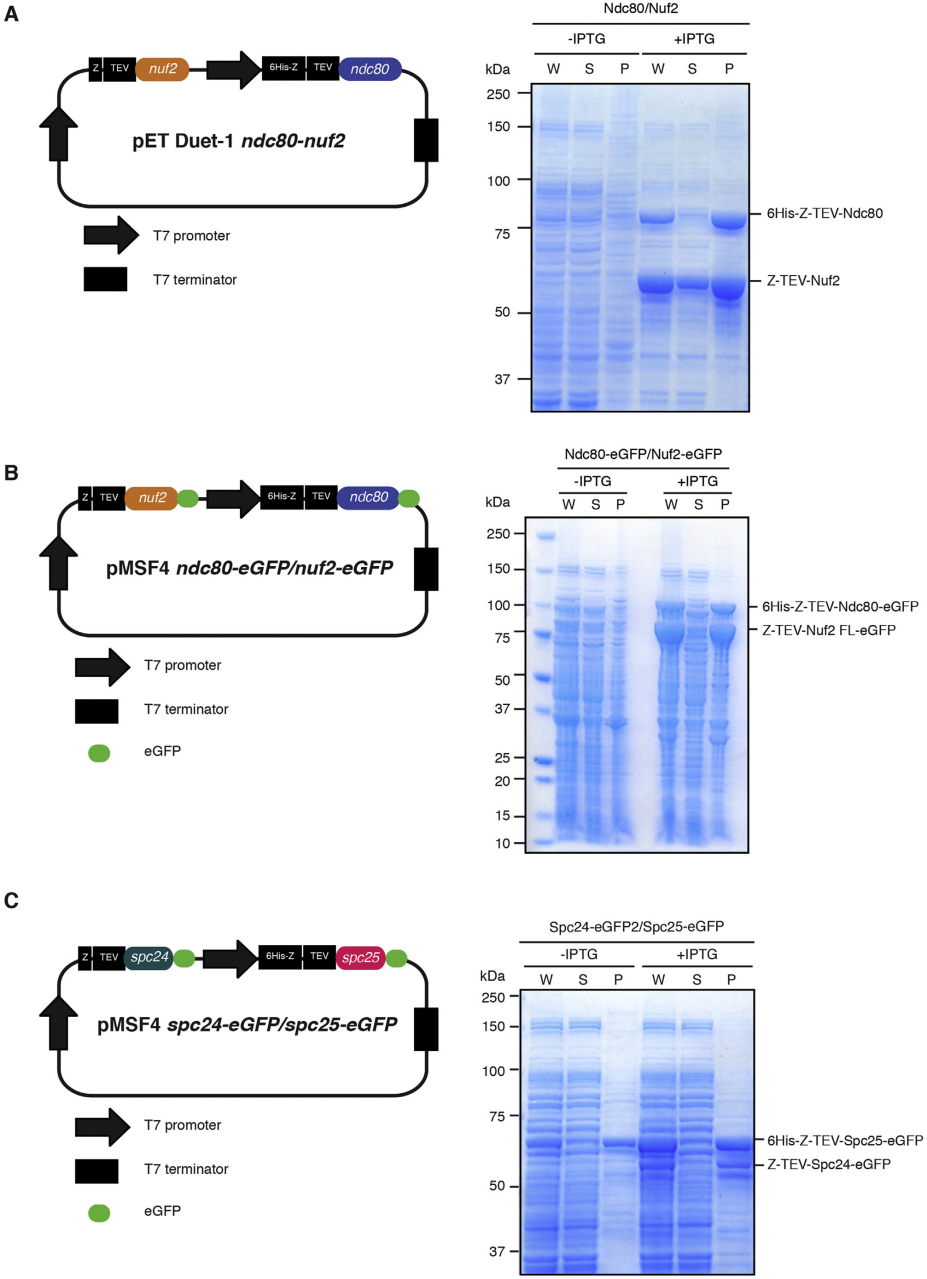
Production of the fission yeast Ndc80-Nuf2 or Spc24-Spc25 sub-complex by bacterial co-expression. **(A)** Schematic diagrams of a plasmid used for the production of the Ndc80-Nuf2 sub-complex (left). 6His-Z-TEV-Ndc80 and Z-TEV-Nuf2 were co-expressed from a bicistronic vector. **(B)** Schematic diagram of the plasmid used for the production of the Ndc80-Nuf2 sub-complex containing C-terminal eGFPs (left). 6His-Z-Ndc80-eGFP and Z-TEV-Nuf2-eGFP were co-expressed from a bicistronic vector. **(C)** Schematic diagram of the plasmid used for the production of the Spc24-Spc25 sub-complex. Z-TEV-Spc24-eGFP and 6His-Z-TEV-Spc25-eGFP were co-expressed from a bicistronic vector. Bacterial whole cell extracts (W), soluble (S) and insoluble fractions (P) before (−) or after (+) 0.1 mM IPTG induction were separated by SDS-PAGE and stained with Coomassie Brilliant Blue (A-C, right).

Plasmids used in this study are as follows: pET-Duet1-6His-Z-TEV-ndc80/Z-TEV-nuf2, pMSF4-6His-Z-TEV-ndc80-eGFP/Z-TEV-nuf2-eGFP, pMSF4-Z-TEV-spc24-eGFP/6His-Z-TEV-spc25-eGFP (Note that Z-TEV and 6His-Z-TEV are omitted from the schematics shown in Fig. 1 for simplicity).

Plasmids producing 6His-Z-TEV-Ndc80/Z-TEV-Nuf2 sub-complex or Z-TEV-Spc24-eGFP/6His-Z-TEV-Spc25-eGFP sub-complex were individually introduced into *E. coli* BL21 (DE3) RIL. Freshly transformed *E.coli* BL21 (DE3) RIL were pre-grown in an LB medium containing ampicillin (100 μg/ml) or kanamycin (50 μg/ml), respectively, at 26 °C for 16 h. The pre-cultures were inoculated into LB medium (3 L) containing ampicillin (100 μg/ml) or kanamycin (50 μg/ml), and the cultures were incubated at 26 °C until the cell density reached an optical density (OD_600_) of 0.5. Then the incubation temperature was changed to 18 °C. After 1 h, IPTG was added to a final concentration of 0.1 mM. The cultures were further incubated at 18 °C for 16 h.

*E. coli* cells expressing Z-TEV-Spc24-eGFP/6His-Z-TEV-Spc25-eGFP sub-complex were then harvested and resuspended in ice-cold Ndc80 buffer (50 mM potassium phosphate (pH7.2), 300 mM KCl, 10%(v/v) glycerol, 2 mM MgCl_2_ and 1 mM β-mercaptoethanol) containing a cocktail of EDTA-free protease inhibitors (Roche) and DNase I (Sigma-Aldrich). These cells were lysed by passing the suspension twice through a high-pressure homogeniser (Emulsiflex C-5: Microfluidics). The cell debris was removed by ultra-centrifugation (50,000 rpm, 30min, 4 °C). Clarified cell lysates were batch incubated for 1 h at 4 °C with 2 g of Protino Ni-TED resin (MACHEREY-NAGEL). After binding, the resin was then packed into an Econo-column (Bio-Rad), and washed with 10 column volumes of Ndc80 buffer. Elution was performed with Ndc80 buffer supplemented with 300 mM imidazole. Eluted proteins were desalted using PD-10 columns (GE Healthcare) equilibrated with Ndc80 buffer. The 6His-Z or Z-tag was cleaved off by incubation with TEV protease overnight at 4 °C. The free 6His-Z, Z-tag and the TEV protease were removed by passing the solution through Protino Ni-TED packed column.

Cells expressing 6His-Z-TEV-Ndc80/Z-TEV-Nuf2 sub-complex were resuspended and protein purification was performed by the same procedure described above except that the TEV cleavage step was omitted. After purification by Protino Ni-TED, the protein yield was approximately 12 mg and 20 mg for Ndc80-Nuf2 and Spc24-Spc25, respectively. Equal amounts (10 mg) of the purified 6His-Z-TEV-Ndc80/Z-TEV-Nuf2 sub-complex and the purified Spc24-eGFP/Spc25-eGFP sub-complex were mixed in Ndc80 buffer and incubated at 4 °C for 16 h, allowing the assembly of the 6His-Z-TEV-Ndc80-eGFP holo complex. This two-step purification procedure is basically the same as that used for the reconstitution and purification of the human Ndc80 holo complex from bacteria used before [16]. The reconstituted 6His-Z-TEV-Ndc80-eGFP complex was further purified by 2 g of Protino Ni-TED resin, followed by TEV cleavage. After the free 6His-Z-tag and the TEV protease were removed, the Ndc80-eGFP holo complex was run on size-exclusion chromatography using a Superose6 10/300 GL columns (GE Healthcare) equilibrated with Ndc80 buffer. The peak fractions containing Ndc80-eGFP holo complex were concentrated to 2 mg/ml by Vivaspin6-50 K (GE Healthcare) and were flash-frozen in liquid nitrogen. The final yield of Ndc80-eGFP holo complex was approximately 1 mg from 3 L culture of *E.coli* expressing Ndc80-Nuf2 or Spc24-Spc25 sub-complex.

The purification of the full-length Dis1 constructs (Dis1 and Dis1-eGFP) and Mal3 was performed as described previously [32], [40]. Porcine brain tubulin was purified with standard procedures and cycled tubulin fractions were labelled with Cy5 (Cyanine5 NHS ester, Lumiprobe) or EZ-link NHS Biotin (Thermo Scientific) as described previously [35]. Protein concentrations were determined by Bradford assay (Bio-Rad), with bovine serum albumin (Sigma-Aldrich) or UV at 280 nm as the standard.

### 2.2 Gel filtration analysis

The purified fraction containing Ndc80-eGFP holo complex was fractionated through a 25 ml Superose 6 10/300 GL column (GE Healthcare) in ice-cold Ndc80 buffer.

The molecular mass markers including Blue dextran 2000 (2000 kDa), thyroglobulin (669 kDa), Ferritin (440 kDa), Aldorase (158 kDa), Conalbumin (75 kDa) (GE Healthcare) were fractionated similarly in the same buffer.

### 2.3 Microtubule cosedimentation assay

Tubulin (120 μM) was polymerised in BRB80 (80 mM PIPES, 1 mM EGTA, 1 mM MgCl_2_, pH6.8 with KOH) supplemented with 1 mM GTP for 20 min at 37 °C. Paclitaxel (taxol (Sigma-Aldrich)) was then added to a final concentration of 160 μM and the taxol-stabilised microtubules were pelleted by centrifugation at 20,800 × g for 5 min at the room temperature. To examine the affinity of the Ndc80 holo complex for the taxol-GDP lattice of microtubules, 0.5 μM Ndc80 holo complex was incubated with various concentrations (0–4 μM) of taxol-stabilised microtubules for 10 min at room temperature in BRB80 supplemented with varied concentrations of KCl and 20 μM taxol. Microtubules and bound Ndc80 holo complex were then pelleted by centrifugation for 10 min at 227,226 × g at 25 °C in an Optima TLX ultracentrifuge (Beckman) using a TLA100 rotor. Supernatant and pellet were immediately separated and treated with SDS/PAGE loading buffer. Samples were run on 3–8% Tris/Acetate gel (Bio-Rad) and stained with Coomassie brilliant blue (Bio-Rad).

The gels were scanned and the intensities of individual protein bands were measured using Image J software (NIH). To measure band intensities, rectangular regions of equal size were drawn around each band and the background-corrected mean pixel intensity was recorded. The precipitated fraction (%) was obtained by dividing the mean intensity of the pellet fraction by the sum of the intensities of the pellet and supernatant fractions.

### 2.4 TIRF microscopy

TIRF microscopy-based dynamic microtubule assays were performed as previously described [40]. Flow chambers were assembled from a biotin-PEG functionalised coverslip attached to a PLL-PEG passivated microscope slide via double-sided sticky tape. Dimly Cy5-labelled microtubules were polymerised from glass immobilised, brightly labelled (33% Cy5-labelled tubulin) GMPCPP (Jena Bioscience)-stabilised microtubule seeds in the presence of proteins of interest. TIRF assay buffer consisted of BRB80 supplemented with 85 mM KCl, 1 mM GTP, 10 mM β-mercaptoethanol, 0.1% Brij-35, 0.1% methylcellulose (4000 cP, Sigma-Aldrich) and an oxygen scavenger system (20 mM glucose, 320 μg/ml glucose oxidase (Serva) and 55 μg/ml catalase (Sigma-Aldrich)).

For simultaneous dual-colour time-lapse imaging of Cy5 and GFP channels, imaging was performed at 1 s intervals with 100 ms exposure time, using a magnification of ×100 at 30 ± 1 °C on a IX81 Olympus inverted microscope equipped with a Cascade II, cooled charge-coupled device camera (Photometrics), illuminating the sample with 488 nm and 640 nm lasers.

Image analysis was performed using Image J software (NIH). Image J was used to create merged images taken with different fluorescence channels (single frames or kymographs) to visualise the localisations of proteins of interest on microtubules. For background subtraction of the images, the Image J rolling ball functionality was used (30 pixel radius rolling ball subtraction and 2 frame running average using the “running z projector” plug in).

## 3 Results and discussion

### 3.1 Co-expression and purification of the recombinant *S. pombe* Ndc80 holo complex

There are two common approaches to produce recombinant protein complexes [41]. One is simultaneous co-expression of all the subunits in an appropriate host cell, allowing a spontaneous assembly of the recombinant complex inside the cell. The other approach relies on the assembly of individually expressed and purified subunits into a complex *in vitro*. We first constructed a plasmid that contains *ORFs* encoding all four Ndc80 complex subunits in a single polycistronic vector designed for expression in *E. coli*. However, for unknown reasons, the stoichiometry of each component in the purified complex was not equal after purification (data not shown).

Therefore, we next constructed two dicistronic plasmids to express the Ndc80-Nuf2 and Spc24-Spc25 sub-complexes separately. Each sub-complex was then purified individually, followed by mixing both sub-complexes, allowing the Ndc80 holo complex to form *in vitro*. However, we found that the reconstituted fission yeast Ndc80-Nuf2 complex was insoluble under several conditions tested; these include different *E. coli* host strains, various culture conditions and fusion tags (i.e. GST and MBP) (data not shown). Nonetheless, we found that Z-tag or 6His-Z [38], [39] to the N terminus of Ndc80 significantly increased the expression and solubility of Ndc80 when co-expressed with the Z-tagged Nuf2 (Fig. 1A). We also observed that the addition of eGFP to the C-terminus of these two proteins did not impair the solubility of the Ndc80-Nuf2 sub-complex (Fig. 1B). To facilitate the removal of the 6His-Z or Z-tag from Ndc80 or Nuf2 respectively, TEV protease cleavage sites were inserted between the 6His-Z (or Z-tag) and Ndc80 (or Nuf2). We also created the similar constructs for the Spc24-Spc25 sub-complex, because non-tagged Spc24 and Spc25 were also found to be insoluble. Like Ndc80 and Nuf2, the fusion of a 6His-Z to the N terminus of Spc25 significantly improved the efficiency of expression and protein solubility, when co-expressed with the Z-tagged Spc24 (Fig. 1C).

**Fig. 2:**
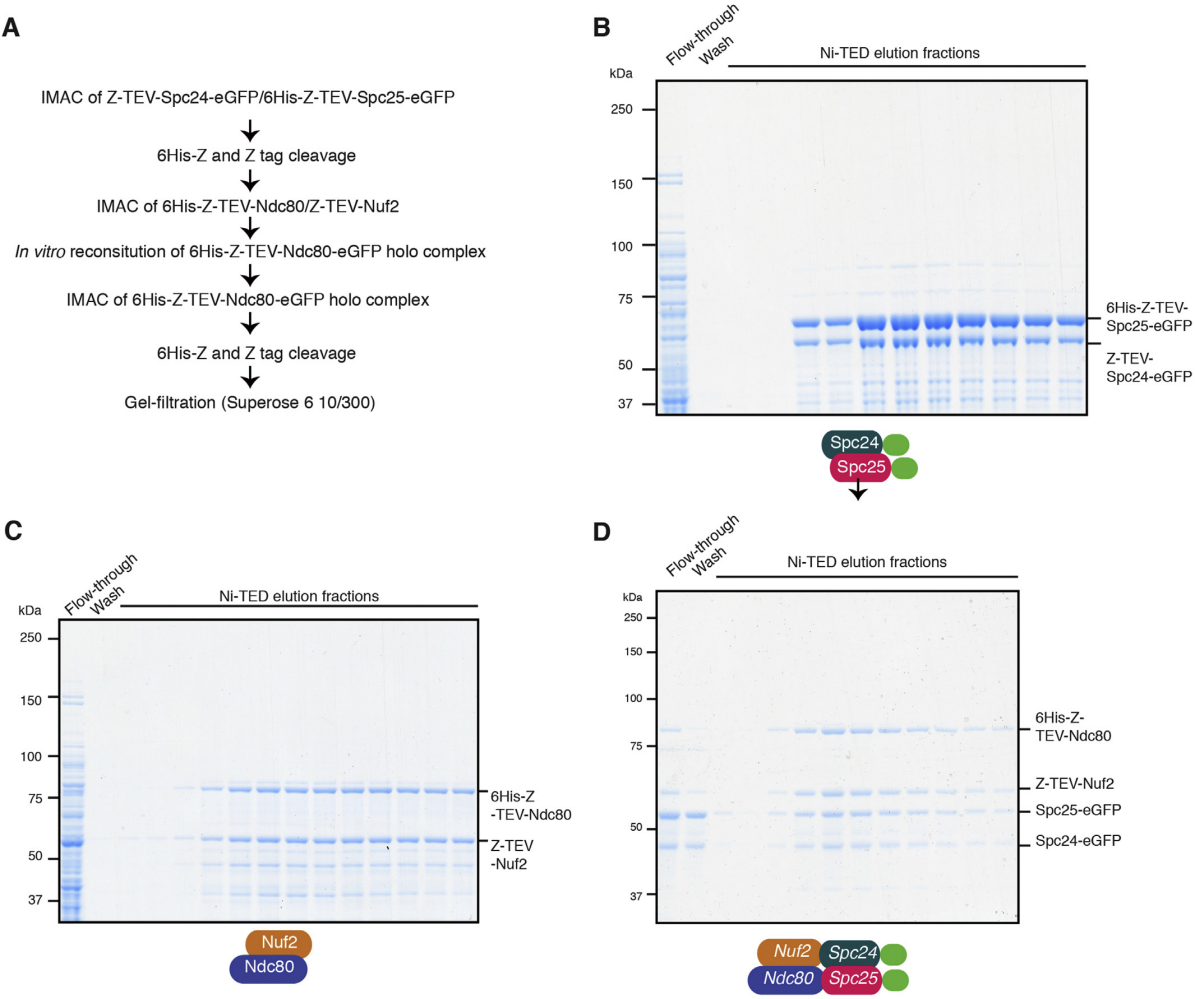
Immobilised metal affinity chromatography of the Ndc80-Nuf2 sub-complex, the Spc24-Spc25 sub-complex and the Ndc80 holo complex. **(A)** A purification flow chart for the Ndc80-eGFP holo complex. **(B)** Elution profiles of the Spc24-Spc25 sub-complex containing the C-terminal eGFPs (6His-Z-TEV-Spc25-eGFP and Z-TEV-Spc24-eGFP) from Ni-TED column. **(C)** Elution profiles of the 6His-Z-TEV-Ndc80 and Z-TEV-Nuf2 sub-complex from Ni-TED column. **(D)** Elution profiles of the 6His-Z-TEV-Ndc80-eGFP holo complex from the Ni-TED column. The holo complex is composed of 6His-Z-TEV-Ndc80, Z-TEV-Nuf2, Spc24-eGFP and Spc25-eGFP. Proteins were stained with Coomassie Brilliant Blue (**B-D**).

**Fig. 3:**
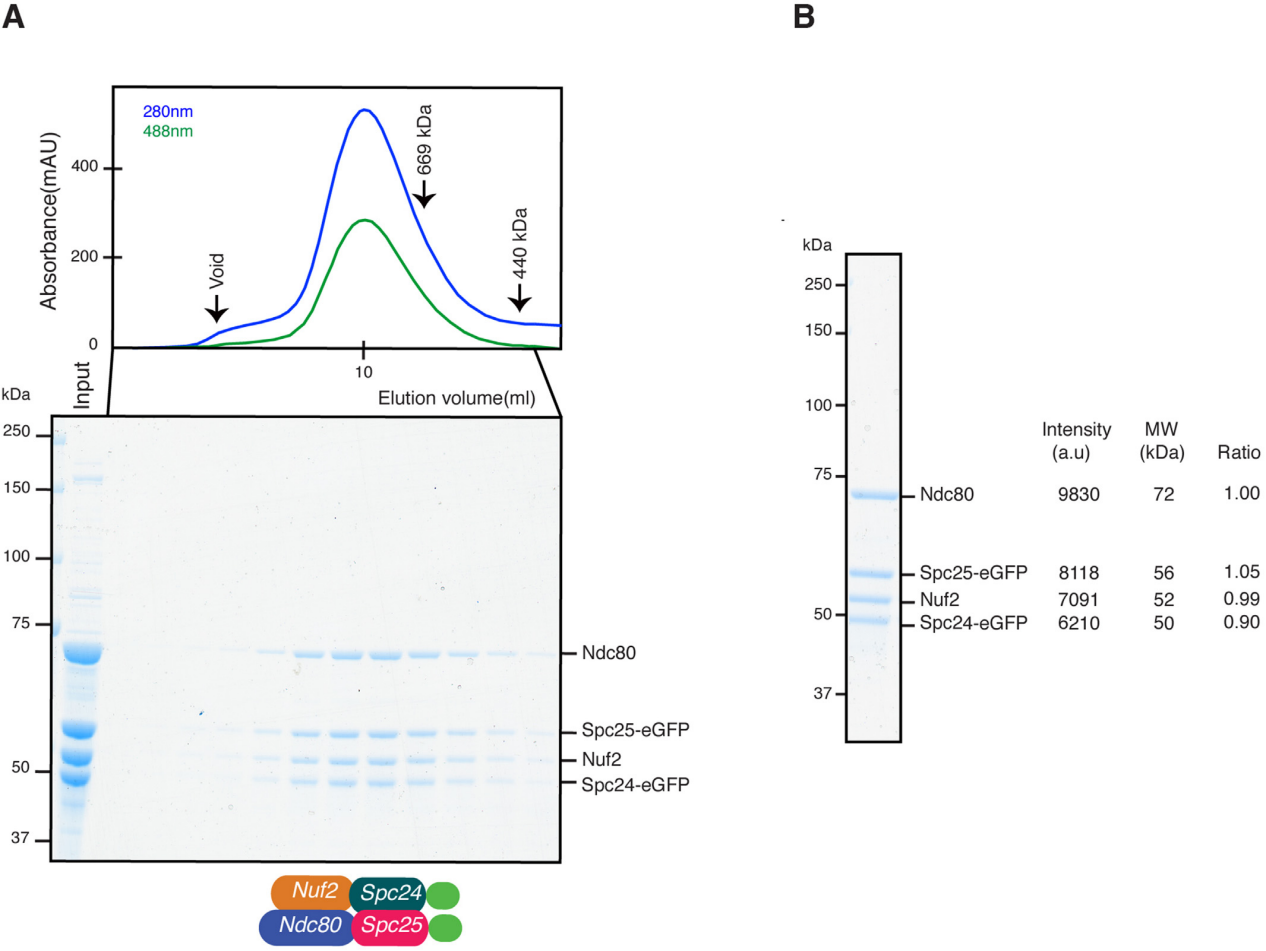
Gel filtration chromatography of the Ndc80-eGFP holo complex. **(A)** Elution profiles measured at the indicated wavelengths of the Ndc80-eGFP holo complex from gel filtration chromatography. The black arrows above the elution profile indicate the positions of the peaks of the molecular mass markers (top). Corresponding patterns of elution fractions on SDS-PAGE (Coomassie Brilliant Blue staining) are shown at the bottom. **(B)** Densitomeric scanning of band intensities corresponding to purified four subunits was performed. Stoichiometry was then calculated; band intensities were divided by individual molecular weights. The value of Ndc80 is assigned as 1.

The *S. pombe* Ndc80 holo complex was purified in the following manner (Fig. 2A). 6His-Z-Spc25/Z-Spc24 and 6His-Z-Ndc80/Z-Nuf2 sub-complexes were eluted separately from a Ni-TED column (Fig. 2B and C). Then, 6His-Z and Z-tag were removed from Spc24 and Spc25, respectively, by TEV protease digestion. Next, the Ndc80 holo complex was allowed to assemble *in vitro* by incubating the 6His-Z-TEV-Ndc80/Z-TEV-Nuf2 sub-complex together with the Spc24-Spc25 sub-complex, and the reconstituted 6His-Z-TEV-Ndc80-eGFP complex was eluted from a Ni-TED column (Fig. 2D). The 6His-Z and Z-tag were then removed by TEV protease digestion. Lastly, the Ndc80 holo complex was further purified by size-exclusion chromatography (Fig. 3A). The final yield of purified Ndc80-eGFP holo complex was approximately 1 mg from 3 L *E. coli* culture.

Accordingly, *S. pombe* Ndc80 (72 kDa), Nuf2 (52 kDa), Spc24-eGFP (50 kDa) and Spc25-eGFP (56 kDa) co-eluted as a single peak (Fig. 3A top). Importantly, an SDS-PAGE gel of the elution fractions indicated that the purified complex is composed of all four subunits with equal stoichiometry (Fig. 3A bottom and 3B). The apparent molecular mass of the complex as estimated from its gel filtration elution profile was over 669 kDa. This was larger than the theoretical molecular mass of the heterotetramer (230 kDa), which is expected for an elongated molecule like the Ndc80 holo complex with its rod-like structure [42]. This shift in the apparent molecular mass agrees with an earlier report showing the same shift for a purified human Ndc80 complex [16].

### 3.2 Physical interaction between the Ndc80 complex and MTs

**Fig. 4:**
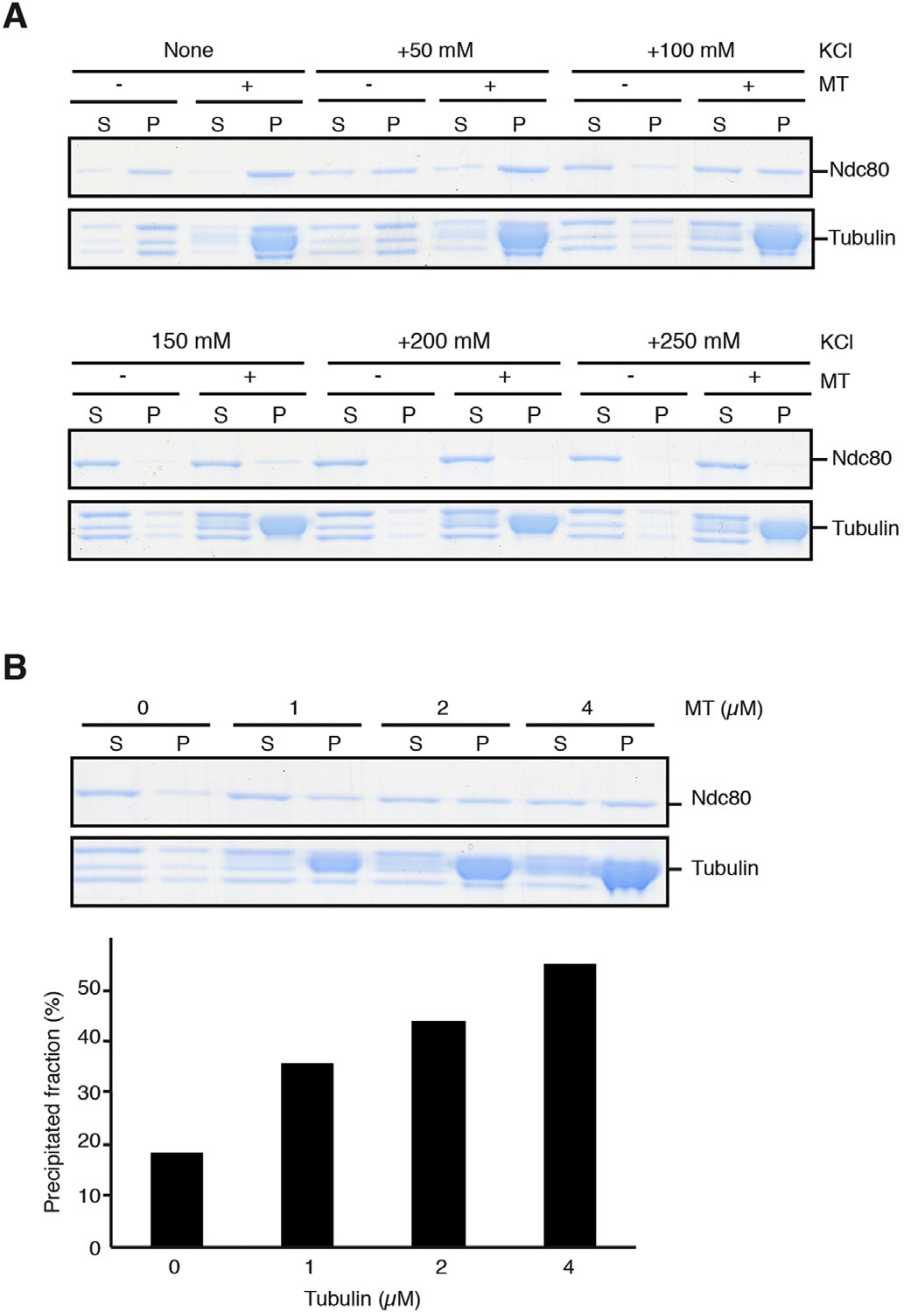
Microtubule-binding activity of the fission yeast Ndc80-eGFP holo complex. **(A)** Microtubule binding activity of the Ndc80-eGFP holo complex was examined by a microtubule cosedimentation assay. The Ndc80-eGFP holo complex or tubulin concentrations were kept constant at 0.5 μM or 2.0 μM respectively, while KCl concentration was varied (0, 50, 100, 150, 200, 250 mM) in BRB80. The mixtures were centrifuged through a cushion buffer and both supernatant (S) and pellet fractions (P) were stained with Coomassie Brilliant Blue. **(B)** Microtubule binding activity. The Ndc80-eGFP holo complex concentrations were kept constant at 0.5 μM, while the tubulin concentration was varied (from the left to the right, 0, 1, 2, 4 μM) for polymerising MTs in BRB80 supplemented with 100 mM KCl. The mixtures were centrifuged through a cushion buffer and both supernatant (S) and pellet fractions (P) were stained with Coomassie Brilliant Blue. The percentage of precipitated fractions of the Ndc80 complex at individual tubulin concentrations is calculated and plotted at the bottom.

Next, we examined the MT binding activity of the recombinant *S. pombe* Ndc80-eGFP holo complex by standard MT cosedimentation assays. In BRB80 buffer, almost all Ndc80-eGFP holo complex fractionated in the pellet even in the absence of MTs, indicating low solubility. However, we found that BRB80 supplemented with 100 mM KCl significantly improved the solubility of the fission yeast Ndc80-eGFP holo complex, and importantly under this condition, the Ndc80 complex cosedimented with polymerised MTs (Fig. 4A). Consistent with a previous study [19], we also found that the interaction between the Ndc80-eGFP holo complex and MTs was sensitive to ionic strength; the MT binding activity was largely lost in the presence of ≥150 mM KCl (Fig. 4A).

Cosedimentation experiments showed that in a BRB80 buffer supplemented with 100 mM KCl, the recombinant Ndc80-eGFP holo complex bound to taxol (paclitaxel)-stabilised MTs in a MT dose-dependent manner (Fig. 4B). Hence, we conclude that the fission yeast Ndc80-eGFP holo complex has an intrinsic MT binding activity as Ndc80 from other organisms [6], [7], [16], [20].

### 3.3 Fission yeast Ndc80 complex binds the microtubule lattice and tracks the growing plus end in the presence of Mal3/EB1

**Fig. 5:**
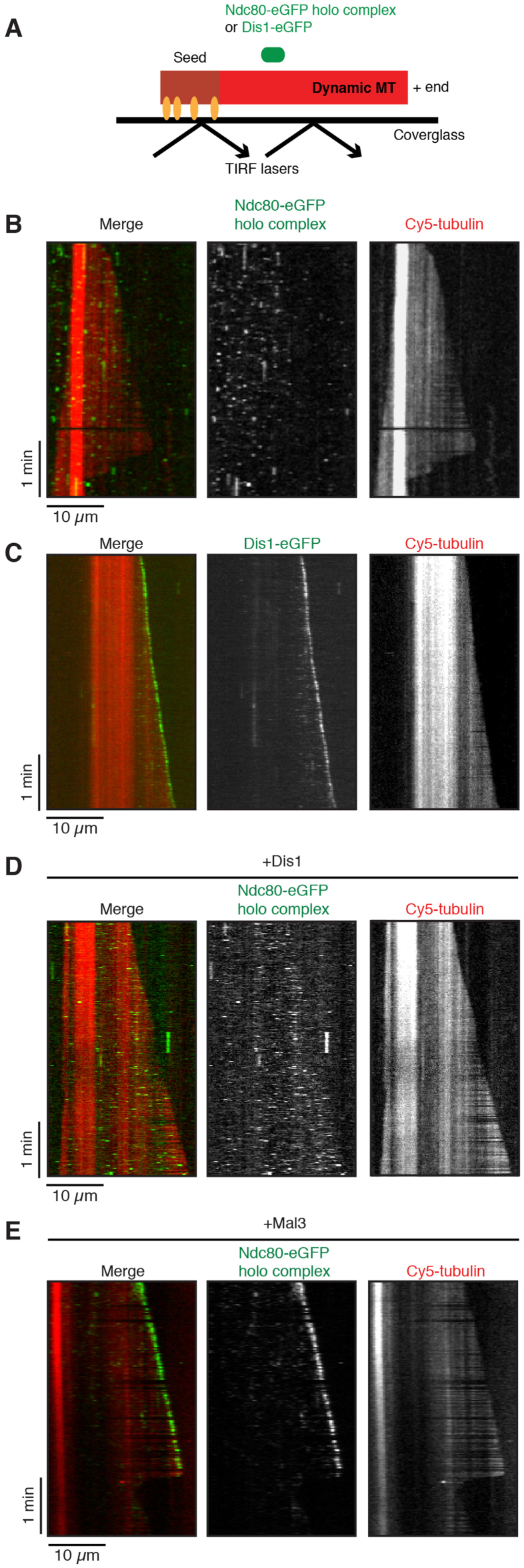
Microtubule-binding properties of the fission yeast Ndc80-eGFP holo complex with TIRF-M assays. **(A)** Scheme of the TIRF microscopy-based dynamic microtubule *in vitro* assay. **(B)** Dual- and single colour TIRF microscopy kymographs of a growing Cy5-microtubule (red in merge) with Ndc80-eGFP holo complex (green in merge) at 100 pM. It is of note that the horizontal dark line seen in the lower part of the Cy5-tubulin panel is unintentionally introduced for unknown reason during the acquisition of the TIRF data. **(C)** Dual- and single colour kymographs of a growing Cy5-microtubule (red in merge) with Dis1-eGFP (green in merge) at 10 nM tracking the growing plus end. **(D)** Kymographs of a Cy5-microtubule (red in merge) with Ndc80-eGFP holo complex (green in merge) at 100 pM in the presence of 10 nM unlabelled Dis1. **(E)** Kymographs of a Cy5-microtubule (red in merge) with Ndc80-eGFP holo complex (green in merge) at 100 pM in the presence of 20 nM unlabelled Mal3. Note that the Ndc80 complex track the growing plus end of the microtubule. Cy5-labelled tubulin concentration was always 8 μM in BRB80 supplemented with 85 mM KCl. Scale bars, 10 μm (horizontal) and 1 min (vertical) **(B-E**). (For interpretation of the references to colour in this figure legend, the reader is referred to the web version of this article.)

We next observed the behaviour of the Ndc80-eGFP holo complex on dynamic MTs under TIRF-microscopy-based *in vitro* assays. MTs were grown from immobilised and GMPCPP-stabilised microtubule seeds (Materials and Methods) in the presence of Cy5-labelled tubulin and GTP [40] (Fig. 5A). This assay indicated that some Ndc80-eGFP holo complexes transiently bound to the lattice of MTs (Fig. 5B). It should be noted that unlike the human complex [20], the *S. pombe* Ndc80-eGFP holo complex could not track disassembling MT ends under the conditions tested (Fig. 5B).

Our previous results indicated that Ndc80 directly or indirectly interacts with the MAP Dis1/chTOG [23] that tracks the plus end of dynamic MTs and possesses a MT polymerase activity as other members of this family [32]. Intriguingly, Dis1 forms a stable complex with the autonomous MT plus-end tracking protein Mal3/EB1, thereby regulating mitotic progression [32]. In addition, it was reported that budding yeast and human orthologues of Dis1, Stu2 and chTOG are capable of directly interacting with the Ndc80 complex [43]. Given these results, we next addressed the question whether Dis1 or Mal3 could alter the localisation of the Ndc80 holo complex on dynamic MTs. Whereas 10 nM purified Dis1-eGFP localised to the growing MT ends (Fig. 5C) as previously shown [32], no significant changes of the localisation of the 100 pM Ndc80-eGFP holo complex occurred in the presence of Dis1 in our TIRF-microscopy assays at physiological ionic strength; Ndc80-eGFP still bound the MT lattice even in the presence of Dis1 (Fig. 5D). Remarkably, on the other hand, the addition of 20 nM Mal3 did alter the localisation pattern of 100 pM Ndc80 inducing MT plus end accumulation (Fig. 5E). This implies that Ndc80 and Dis1 might interact through Mal3 at the kinetochore-microtubule interface, thereby modulating MT dynamics. Further work is necessary to explore this proposition.

In this study, we have established a purification protocol of the fission yeast Ndc80 holo complex using a bacterial expression system. We have found that the Ndc80 complex binds the MT lattice; interestingly, Mal3 is capable of localising the Ndc80 complex to the growing MT plus end. As the *in vivo* kinetochore-microtubule interface comprises a complex structure with a cohort of regulatory factors, more work awaits in order to investigate the molecular mechanism by which the spindle microtubule attaches to the kinetochore and ensures high-fidelity chromosome segregation. Both Mal3 and the Ndc80 complex interact with other MAPs; these include Dis1/chTOG [23], the Alp7/TACC-Alp14/chTOG complex [33], the Klp5-Klp6 kinesin-8 complex [34], the Dam1 complex [23], [44] and the Klp2 minus end-directed kinesin-14 [45]. How these proteins orchestrate MT dynamics at the kinetochore-microtubule interface is a major issue to be explored. By exploiting the *in vitro* system developed in this study, recapitulating the dynamic MT behaviour in the presence of the Ndc80 holo complex and other MAPs is a major goal of future investigations.

## Author contributions

Y.M., S.P.M., T.S and T.T. designed the experiments. M.Y. performed most experiments. S.P.M. performed initial protein purifications and TIRF-microscopy experiments. Y.M. and T.T. wrote the manuscript helped by input from S.P.M., T.S.

## Competing interests

The authors declare that they have no conflict of interest.
